# Crystal structure of BaMn_2_(AsO_4_)_2_ containing discrete [Mn_4_O_18_]^28−^ units

**DOI:** 10.1107/S2056989017016152

**Published:** 2017-11-14

**Authors:** Salvador Alcantar, Hollis R. Ledbetter, Kulugammana G. S. Ranmohotti

**Affiliations:** a Division of Science, Mathematics and Technology, Governors State University, 1 University Parkway, University Park, IL 60484-0975, USA

**Keywords:** orthoarsenate, tetra­meric units, layered framework stucture, bond-valence sum calculations, crystal structure

## Abstract

In our attempt to search for mixed alkaline-earth and transition metal arsenates, the title compound, barium dimanganese(II) bis­(arsenate), has been synthesized by employing a high-temperature RbCl flux. The crystal structure of BaMn_2_(AsO_4_)_2_ is made up of MnO_6_ octa­hedra and AsO_4_ tetra­hedra assembled by sharing corners and edges into infinite slabs with composition [Mn_2_(AsO_4_)_2_]^2−^ that extend parallel to the *ab* plane. The barium cations reside between parallel slabs maintaining the inter­slab connectivity through coordination to eight oxygen anions. The layered anionic framework comprises weakly inter­acting [Mn_4_O_18_]^28−^ tetra­meric units. In each tetra­mer, the manganese(II) cations are in a planar arrangement related by a center of inversion. Within the slabs, the tetra­meric units are separated from each other by 6.614 (2) Å (Mn⋯Mn distances). The title compound has isostructural analogues amongst synthetic Sr*M*_2_(*X*O_4_)_2_ compounds with *M* = Ni, Co, and *X* = As, P.

## Abstract 2

BaMn_2_(AsO_4_)_2_ was isolated from a high-temperature halide flux. Its crystal structure is characterized by infinite sheets made up of AsO_4_ units and distorted MnO_6_ octa­hedra while barium cations inter­leave successive sheets. The layered framework comprises weakly inter­acting [Mn_4_O_18_]^28−^ tetra­meric units. These units in the neighboring layer are separated from each other by 6.614 (2) Å (Mn**⋯**Mn distance).

## Chemical context   

Compounds of vanadates, phosphates, and arsenates with the general formula *AM*_2_(*X*O_4_)_2_, where *A* = Pb or an alkaline earth metal, *M* = Mg or a divalent first row transition metal, and *X* = V, P or As, can adopt different structure types. They have attracted much attention in solid-state physics due to magnetic ordering at low temperatures and the occurrence of (multiple) phase transitions. For *AM*_2_(*X*O_4_)_2_ compounds with Pb or an alkaline earth metal ion on the *A* ^2+^ site and a transition metal with partially filled 3*d* orbitals on the *M* ^2+^ site, one-dimensional magnetic properties with high anisotropy and weak inter­chain inter­actions have been reported (Bera *et al.*, 2013 ▸). The crystal structures of some of these compounds comprise screw-chains made up of *M*O_6_ octa­hedra, separated by non-magnetic VO_4_ (V^5+^; 3*d* ^0^) tetra­hedra, resulting in a quasi one-dimensional structure. Five representatives of this family have been characterized crystallographically, *viz*. BaMg_2_(VO_4_)_2_ (Velikodnyi *et al.*, 1982 ▸), BaCo_2_(VO_4_)_2_ (Wichmann & Müller-Buschbaum, 1986*a* ▸), BaMn_2_(VO_4_)_2_, BaMgZn(VO_4_)_2_ and Ba_1/2_Sr_1/2_Ni_2_(VO_4_)_2_ (Von Postel & Müller-Buschbaum, 1992 ▸). They crystallize in the tetra­gonal crystal system in space group *I*4_1_/*acd* (No. 142). For the related compounds SrMn_2_(VO_4_)_2_ (Niesen *et al.*, 2011 ▸), SrCo_2_(VO_4_)_2_ (Osterloh & Müller-Buschbaum, 1994*a* ▸), PbCo_2_(VO_4_)_2_ (He *et al.*, 2007 ▸) and PbNi_2_(VO_4_)_2_ (Uchiyama *et al.*, 1999 ▸), it was found that they adopt the SrNi_2_(VO_4_)_2_ structure type (Wichmann & Müller-Buschbaum, 1986*b* ▸), crystallizing in space group *I*4_1_ *cd* (No.110), a subgroup of the latter. The only copper(II) vanadate compound with an *AM*_2_(*X*O_4_)_2_ composition is BaCu_2_(VO_4_)_2_ (Vogt & Müller-Buschbaum, 1990 ▸). The crystal structure is also tetra­gonal but belongs to space group *I* 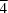
2*d* (No. 122), another subgroup of *I*4_1_/*acd*. BaNi_2_(VO_4_)_2_ (Rogado *et al.*, 2002 ▸) adopts a different structure type as it belongs to the rhombohedral space group *R* 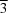 (No. 148) and represents the only quasi-two-dimensional system within the above-mentioned vanadates.

Phosphates containing transition metals have been widely investigated because of their variety of potential applications. They can adopt a plethora of different structure types and show various magnetic properties. With respect to the *AM*_2_(*X*O_4_)_2_ family of compounds, the phosphates BaCu_2_(PO_4_)_2_ (Moqine *et al.*, 1993 ▸), *α*-SrCo_2_(PO_4_)_2_ (El Bali *et al.*, 1993*a* ▸), *β*-SrCo_2_(PO_4_)_2_ (Yang *et al.*, 2016 ▸ **)**, SrNi_2_(PO_4_)_2_ (El Bali *et al.*, 1993*b* ▸), SrNiZn(PO_4_)_2_ (El Bali *et al.*, 2004 ▸) and SrMn_2_(PO_4_)_2_ (El Bali *et al.*, 2000 ▸) crystallize in space group *P* 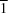 (No. 2). SrCu_2_(PO_4_)_2_ (Belik *et al.*, 2005 ▸) and PbCu_2_(PO_4_)_2_ (Belik *et al.*, 2006 ▸) are isotypic and crystallize in the ortho­rhom­bic crystal system [space group *Pccn* (No. 56)]. The crystal structures of BaNi_2_(PO_4_)_2_ (Čabrić *et al.*, 1982 ▸), and BaFe_2_(PO_4_)_2_ (Kabbour *et al.*, 2012 ▸) possess trigonal symmetry in space group *R* 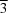 (No. 148). BaCo_2_(PO_4_)_2_, in particular, can exist in several polymorphs such as the rhombohedral *γ*-phase [(*R* 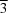 (No. 148); Bircsak & Harrison, 1998 ▸], the monoclinic *α*-phase [*P*2_1_/*a* (No. 14)] and the trigonal *β*-phase [*P* 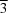 (No. 147); David *et al.*, 2013 ▸], depending on the synthetic conditions and thermal history. It has been reported that *α*-SrZn_2_(PO_4_)_2_ (Hemon & Courbion, 1990 ▸) and SrFe_2_(PO_4_)_2_ (Belik *et al.*, 2001 ▸) adopt different structure types and crystallize in the monoclinic space group *P*2_1_/*c* (No. 14).

Thus far, compared to vanadates and phosphates, only a few arsenates of the *AM*_2_(*X*O_4_)_2_ family have been studied, *viz.* BaNi_2_(AsO_4_)_2_ (Eymond *et al.*, 1969*a* ▸), BaMg_2_(AsO_4_)_2_ and BaCo_2_(AsO_4_)_2_ (Eymond *et al.*, 1969*b* ▸) in space group *R* 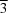, and SrCo_2_(AsO_4_)_2_ (Osterloh & Müller-Buschbaum, 1994*a* ▸) and BaCu_2_(AsO_4_)_2_ (Osterloh & Müller-Buschbaum, 1994*b* ▸) in space groups *P* 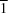 and *P*2_1_/*n*, respectively. To extend our knowledge of the *AM*_2_(*X*O_4_)_2_ system, we have undertaken an investigation of the BaO/MnO/As_2_O_5_ phase diagram and employed a flux method for crystal growth. The present work deals with the determination of the crystal structure of a new mixed-metal orthoarsenate, BaMn_2_(AsO_4_)_2_.

## Structural commentary   

**Figure 1:**
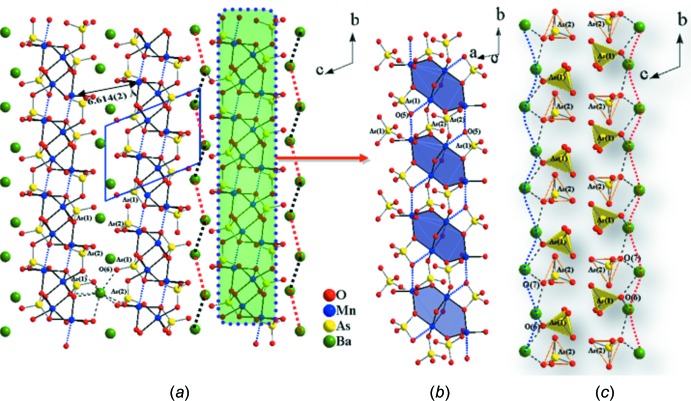
(*a*) Perspective view of the crystal structure of BaMn_2_(AsO_4_)_2_ viewed along the *a* axis. The quasi-two-dimensional lattice is characterized by [Mn_2_(AsO_4_)_2_]^2−^ slabs, which are highlighted by dark- and light-colored lines representing the Mn—O and As—O bonds, respectively. The wavy and dotted lines (right) indicate the zigzag arrays of barium cations. Only one barium cation with bonds is drawn for clarity, demonstrating the function of Ba—O bonds with regard to holding neighboring [Mn_2_(AsO_4_)_2_]^2−^ slabs. (*b*) Part of the crystal structure showing corner- and edge-sharing MnO_6_ octa­hedra and AsO_4_ tetra­hedra. In each tetra­mer, the manganese(II) atoms are in a planar configuration related by a center of inversion. (*c*) Polyhedral representation showing the alternating arrangement of isolated arsenate units. Only two bonds (dotted lines) around barium are shown for clarity.

**Figure 2:**
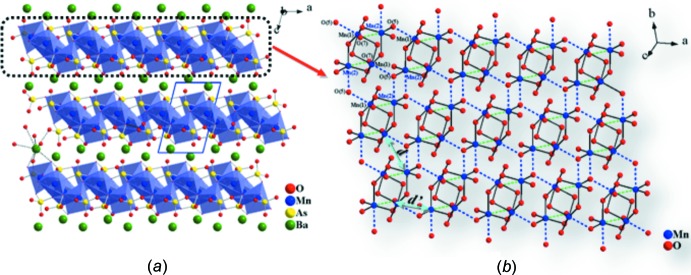
(*a*) Quasi-two-dimensional structure of BaMn_2_(AsO_4_)_2_ shown by polyhedral and ball-and-stick drawing viewed along the *b* axis. (*b*) Ball-and-stick drawing of a portion of the manganese oxide network formed by inter­connected tetra­meric units. Dashed lines represent long Mn—O bonds.

**Figure 3:**
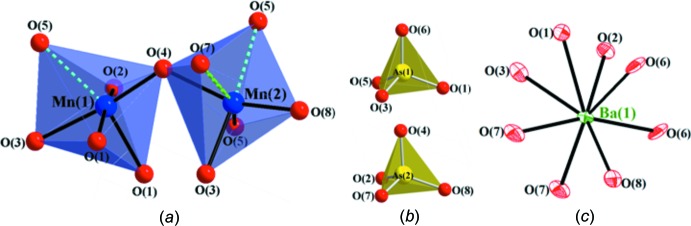
(*a*) Part of the crystal structure showing Mn1O_6_ and Mn2O_6_ octa­hedra sharing corners (polyhedral drawing). To distinguish the two types of bonds (short and long), one is highlighted with solid Mn—O bonds (short) and the other in dotted bonds (long). (*b*) The polyhedral units represent arsenic-centered oxygen tetra­hedra. (*c*) The barium cation resides in a BaO_8_ environment. Displacement ellipsoids represent the 95% probability level.

Besides Ba_2_Mn(AsO_4_)_2_ (Adams *et al.*, 1996 ▸), BaMn_2_(AsO_4_)_2_ represents the second compound to be structurally characterized in the system BaO/MnO/As_2_O_5_. BaMn_2_(AsO_4_)_2_ is isotypic with *β*-SrCo_2_(PO_4_)_2_ (Yang *et al.*, 2016 ▸), SrCo_2_(AsO_4_)_2_ (Osterloh & Müller-Buschbaum, 1994*a* ▸) and SrNi_2_(PO_4_)_2_ (El Bali *et al.*, 1993*b* ▸) (for numerical data for these structures, see Supplementary Table 1 ▸ in the *Supporting information*). The crystal structure of BaMn_2_(AsO_4_)_2_ can be described as a three-dimensional framework containing slabs of composition [Mn_2_(AsO_4_)_2_]^2−^ that are built up from two different MnO_6_ and two different AsO_4_ polyhedra (Fig. 1 ▸) and extend parallel to the *ab* plane (Fig. 2 ▸). Mn1 possesses a distorted octa­hedral coordination environment and exhibits five normal Mn—O bonds and one long Mn—O bond. Mn2 is also six-coordinated and has two long Mn—O bonds, again forming a distorted MnO_6_ octa­hedron (Table1, Fig. 3 ▸ *a*). Similar distortions in MnO_6_ octa­hedra have been observed previously (Adams *et al.*, 1996 ▸; Weil & Kremer, 2017 ▸). The two arsenic atoms are part of AsO_4_ tetra­hedra (Fig. 3 ▸ *b*), with As—O bond lengths ranging from 1.663 (5)–1.710 (4) Å (Table 1 ▸) and O—As—O bond angles from 99.8 (2)–114.6 (2)°. The average As—O bond length (1.688 Å) in the title compound is identical to those of previously reported arsenates (Ulutagay-Kartin *et al.*, 2003 ▸). The bond lengths are also consistent with the sum of the Shannon crystal radii (Shannon, 1976 ▸), 1.68 Å, of four-coordinate As^5+^ (0.475 Å) and two-coordinate O^2−^ (1.21 Å). The barium cations reside between parallel slabs and maintain the inter­slab connectivity through coordination to eight oxygen anions (Fig. 3 ▸ *c*). The average Ba—O bond length, 2.83 Å, matches closely with 2.77 Å, the sum of the Shannon radii for eight-coordinate Ba^2+^ (1.56 Å) and two-coordinated O^2−^ (1.21 Å) ions, and is in agreement with those of other barium arsenates (Weil, 2016 ▸).

**Figure 4:**
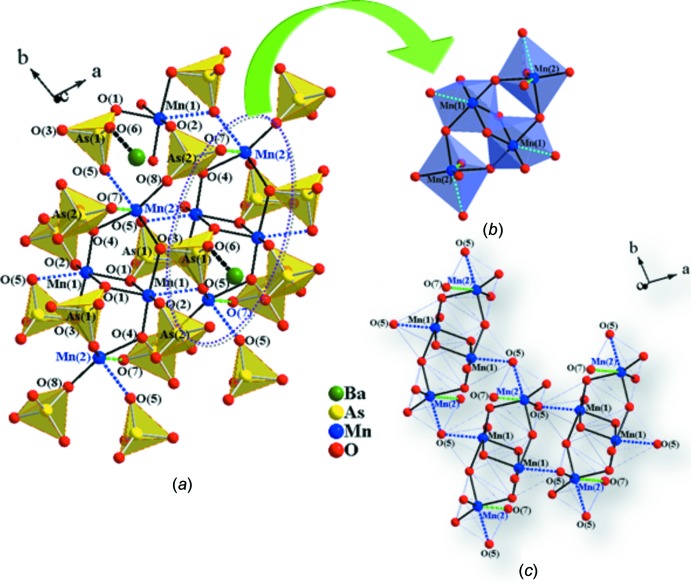
(*a*) The ball-and-stick and polyhedral composite representation showing parts of the Mn—As—O framework. The polyhedral units represent AsO_4_ tetra­hedra. One of the [Mn_4_O_18_]^28−^ units is located in the area outlined by a dotted ellipsoid. The only unshared oxygen atom, O6 of the As1O_4_ tetra­hedra, forms a bond with a Ba cation. (*b*) A tetra­meric unit formed by two edge-sharing Mn1O_6_ octa­hedra and two corner-sharing Mn2O_6_ octa­hedra. (*c*) Three [Mn_4_O_18_]^28−^ units showing the inter­tetra­mer inter­action through long Mn1—O5 and Mn2—O5 bonds (dotted lines).

Fig. 4 ▸ *a* shows two Mn1O_6_ octa­hedra sharing a common edge, O1^v^—O1^vii^ (symmetry codes refer to Table 1 ▸) to form a Mn_2_O_10_ unit with an Mn1**⋯**Mn1 separation of 3.1854 (17) Å and an Mn1—O1—Mn1 angle of 93.34 (18)°. Mn2O_6_ octa­hedra share corners with the Mn_2_O_10_ unit through O3 and O4, resulting in a tetra­meric [Mn_4_O_18_]^28−^ unit (Fig. 4 ▸ *b*). These [Mn_4_O_18_]^28−^ units are inter­linked through AsO_4_ tetra­hedra to give slabs with overall composition [Mn_2_(AsO_4_)_2_]^2−^. Each [Mn_4_O_18_]^28−^ unit inter­acts weakly by sharing oxygen vertices with six other units, whereby the tetra­meric units are separated from each other along the *b* axis by 4.2616 (19) Å [Mn1⋯Mn2(1 − *x*, −*y*, 2 − *z*)] and along the *a* axis by 3.490 (18) Å [Mn1⋯Mn2(*x*, −1 + *y*, −1 + z)]. The distance between the Mn atoms of adjacent slabs [Mn2⋯Mn2(−*x*, 1 − *y*, −*z*)] is 6.614 (2) Å (Fig. 1 ▸ *a*).

As shown in Fig. 4 ▸ *a*, the roles of the two arsenate groups are different. As1O_4_ tetra­hedra share oxygen atoms O1 with Mn1O_6_ octa­hedra, and O3 and O5 atoms with Mn1O_6_ and Mn2O_6_ octa­hedra while oxygen atom O6 points towards neighboring slabs to form a bond with a Ba^2+^ cation (Fig. 4 ▸ *a*). As2O_4_ tetra­hedra, on the other hand, share an edge (O4–O7) with Mn2O_6_ octa­hedra of one tetra­meric unit and share two corners (O7 and O8) with Mn1O_6_ and Mn2O_6_ octa­hedra of two other neighboring tetra­meric units. Thus As1O_4_ and As2O_4_ tetra­hedra inter­link two and three neighboring tetra­meric units, respectively. As shown in Fig. 1 ▸ *c*, As1O_4_ and As2O_4_ tetra­hedra alternate along the *b* axis, and this template-like arrangement allows the barium cations to propagate in a zigzag fashion to maintain the distance between the [Mn_2_(AsO_4_)_2_]^2−^ slabs.

Bond-valence sum (BVS) calculations (Brese & O’Keefe, 1991 ▸) for BaMn_2_(AsO_4_)_2_ result in values of 2.19, 1.84, 4.87, 5.05 and 1.98 valence units for Mn1, Mn2, As1, As2 and Ba1, respectively, which in each case is close to the expected values of 2 for Mn, 5 for As and 2 for Ba.

It is important to note that the barium cations reside in the gaps between adjacent [Mn_2_(AsO_4_)_2_]^2−^ slabs. The large inter-slab separation [6.614 (2) Å] leads us to believe that magnetic inter­actions that occur between these slabs are expected to be extremely weak, and the dominant magnetic exchange is expected to appear between Mn^2+^ ions in the tetra­meric units within a slab. Judging from the reported magnetic properties for related Ba*M*_2_(*X*O_4_)_2_ (*M* = Co, Ni; *X* = As, P) compounds with the magnetic ions sitting on a honeycomb lattice (Martin *et al.*, 2012 ▸ **)**, or those of *β*-SrCo_2_(PO_4_)_2_ (Yang *et al.*, 2016 ▸) and SrNi_2_(PO_4_)_2_ (He *et al.*, 2008 ▸), we also expect inter­esting magnetic phenomena for BaMn_2_(AsO_4_)_2_.

## Synthesis and crystallization   

**Figure 5:**
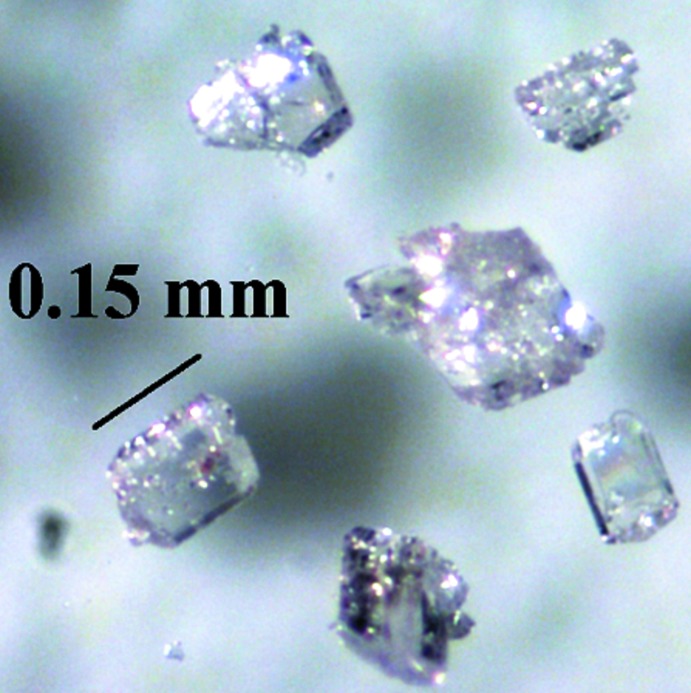
Single crystals of BaMn_2_(AsO_4_)_2_ obtained from a RbCl flux.

Light-pink crystals of BaMn_2_(AsO_4_)_2_ were grown by employing an RbCl flux in a fused silica ampoule under vacuum. MnO (3.81 mmol, 99.999+%, Alfa), BaO (1.90 mmol, 99.99+%, Aldrich) and As_2_O_5_ (1.90 mmol, 99.9+%, Strem) were mixed and ground with RbCl (1:3 by weight) in a nitro­gen-blanketed drybox. The resulting mixture was heated to 818 K at 1 K min^−1^, isothermed for two days, heated to 1023 K at 1 K min^−1^, isothermed for another four days, then slowly cooled to 673 K at 0.1 K min^−1^, followed by furnace-cooling to room temperature. Prismatic crystals of BaMn_2_(AsO_4_)_2_ (Fig. 5 ▸) were retrieved upon washing off recrystallized RbCl with deionized water.

## Refinement   

Crystal data, data collection and structure refinement details are summarized in Table 2 ▸. The final Fourier difference synthesis showed the maximum residual electron density 0.96 Å from Ba1 and the minimum 0.83 Å from the same site.

## Supplementary Material

Crystal structure: contains datablock(s) I. DOI: 10.1107/S2056989017016152/wm5420sup1.cif

Structure factors: contains datablock(s) I. DOI: 10.1107/S2056989017016152/wm5420Isup2.hkl

Supporting information file. DOI: 10.1107/S2056989017016152/wm5420sup3.pdf

CCDC reference: 1584656

Additional supporting information:  crystallographic information; 3D view; checkCIF report

## Figures and Tables

**Table 1:** Selected bond lengths (Å)

Mn1—O4^i^	2.052 (5)	Mn2—O7	2.518 (5)
Mn1—O2^ii^	2.108 (5)	Mn2—O5^vi^	2.526 (5)
Mn1—O1^iii^	2.179 (4)	As1—O6	1.663 (5)
Mn1—O1^iv^	2.200 (5)	As1—O1	1.697 (5)
Mn1—O3	2.202 (5)	As1—O5	1.709 (5)
Mn1—O5	2.491 (5)	As1—O3	1.710 (4)
Mn2—O8^v^	2.094 (5)	As2—O7	1.669 (5)
Mn2—O4	2.136 (5)	As2—O2	1.677 (5)
Mn2—O5^iv^	2.152 (5)	As2—O8	1.677 (4)
Mn2—O3	2.178 (5)	As2—O4	1.698 (5)

Symmetry codes: (i) 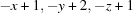; (ii) 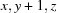; (iii) 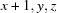; (iv) 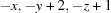; (v) 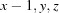; (vi) 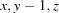.

**Table 2:** Experimental details

Crystal data	
Chemical formula	BaMn_2_(AsO_4_)_2_
*M*_r_	525.06
Crystal system, space group	Triclinic, *P* 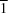
Temperature (K)	293
*a*, *b*, *c* (Å)	5.7981 (12), 7.0938 (14), 9.817 (2)
α, β, γ (°)	109.75 (3), 100.42 (3), 98.40 (3)
*V* (Å^3^)	364.26 (15)
*Z*	2
Radiation type	Mo *K*α
μ (mm^−1^)	17.78
Crystal size (mm)	0.20 × 0.10 × 0.06

Data collection	
Diffractometer	Rigaku AFC8S
Absorption correction	Multi-scan (*REQAB*; Rigaku, 1998 ▸)
*T*_min_, *T*_max_	0.808, 1.000
No. of measured, independent and observed [*I* > 2σ(*I*)] reflections	3149, 1330, 1254
*R*_int_	0.035
(sin θ/λ)_max_ (Å^−1^)	0.606

Refinement	
*R*[*F* ^2^ > 2σ(*F* ^2^)], *wR*(*F* ^2^), *S*	0.039, 0.097, 1.14
No. of reflections	1330
No. of parameters	119
Δρ_max_, Δρ_min_ (e Å^−3^)	3.25, −2.93

Computer programs: *CrystalClear* (Rigaku, 2006 ▸), *SHELXT* (Sheldrick, 2015*a* ▸), *SHELXL2014* (Sheldrick, 2015*b* ▸), *DIAMOND* (Brandenburg, 1999 ▸) and *publCIF* (Westrip, 2010 ▸).

## supplementary crystallographic information

### Crystal data

**Table:**

BaMn_2_(AsO_4_)_2_	*Z* = 2
*M**_r_* = 525.06	*F*(000) = 472
Triclinic, *P*1	*D*_x_ = 4.787 Mg m^−^^3^
*a* = 5.7981 (12) Å	Mo *K*α radiation, λ = 0.71073 Å
*b* = 7.0938 (14) Å	Cell parameters from 3100 reflections
*c* = 9.817 (2) Å	θ = 2.3–25.2°
α = 109.75 (3)°	µ = 17.78 mm^−^^1^
β = 100.42 (3)°	*T* = 293 K
γ = 98.40 (3)°	Column, light pink
*V* = 364.26 (15) Å^3^	0.20 × 0.10 × 0.06 mm

### Data collection

**Table:**

Rigaku AFC8S diffractometer	1254 reflections with *I* > 2σ(*I*)
Radiation source: fine-focus sealed tube	*R*_int_ = 0.035
φ and ω scans	θ_max_ = 25.5°, θ_min_ = 2.3°
Absorption correction: multi-scan (*REQAB*; Rigaku, 1998)	*h* = −6→7
*T*_min_ = 0.808, *T*_max_ = 1.000	*k* = −8→8
3149 measured reflections	*l* = −11→11
1330 independent reflections	1 standard reflections every 1 reflections

### Refinement

**Table:**

Refinement on *F*^2^	Primary atom site location: structure-invariant direct methods
Least-squares matrix: full	Secondary atom site location: difference Fourier map
*R*[*F*^2^ > 2σ(*F*^2^)] = 0.039	*w* = 1/[σ^2^(*F*_o_^2^) + (0.0674*P*)^2^ + 0.2802*P*] where *P* = (*F*_o_^2^ + 2*F*_c_^2^)/3
*wR*(*F*^2^) = 0.097	(Δ/σ)_max_ = 0.001
*S* = 1.14	Δρ_max_ = 3.25 e Å^−^^3^
1330 reflections	Δρ_min_ = −2.93 e Å^−^^3^
119 parameters	Extinction correction: SHELXL2014 (Sheldrick, 2015a), Fc^*^=kFc[1+0.001xFc^2^λ^3^/sin(2θ)]^-1/4^
0 restraints	Extinction coefficient: 0.076 (3)

### Special details

**Table:**

Geometry. All esds (except the esd in the dihedral angle between two l.s. planes) are estimated using the full covariance matrix. The cell esds are taken into account individually in the estimation of esds in distances, angles and torsion angles; correlations between esds in cell parameters are only used when they are defined by crystal symmetry. An approximate (isotropic) treatment of cell esds is used for estimating esds involving l.s. planes.

### Fractional atomic coordinates and isotropic or equivalent isotropic displacement parameters (Å^2^)

**Table:**

	*x*	*y*	*z*	*U*_iso_*/*U*_eq_	
Ba1	0.24355 (7)	0.79849 (6)	0.05371 (4)	0.0100 (2)	
Mn1	0.36901 (17)	1.15237 (15)	0.44407 (11)	0.0091 (3)	
Mn2	0.00727 (18)	0.59526 (15)	0.35472 (12)	0.0105 (3)	
As1	−0.15775 (11)	1.02502 (10)	0.30239 (7)	0.0061 (3)	
As2	0.40017 (11)	0.42782 (10)	0.24010 (7)	0.0062 (3)	
O1	−0.3934 (8)	0.9502 (7)	0.3669 (5)	0.0092 (10)	
O2	0.3546 (8)	0.1879 (7)	0.2382 (5)	0.0124 (10)	
O3	0.0571 (8)	0.8910 (7)	0.3296 (5)	0.0105 (10)	
O4	0.3801 (8)	0.5917 (7)	0.4075 (5)	0.0099 (10)	
O5	−0.0125 (8)	1.2729 (7)	0.4122 (5)	0.0125 (10)	
O6	−0.2437 (9)	0.9808 (7)	0.1213 (5)	0.0139 (11)	
O7	0.1684 (8)	0.4587 (7)	0.1276 (6)	0.0133 (11)	
O8	0.6676 (8)	0.4888 (7)	0.2047 (5)	0.0127 (10)	

### Atomic displacement parameters (Å^2^)

**Table:**

	*U*^11^	*U*^22^	*U*^33^	*U*^12^	*U*^13^	*U*^23^
Ba1	0.0116 (3)	0.0073 (3)	0.0072 (3)	−0.00194 (18)	0.00103 (18)	0.0003 (2)
Mn1	0.0061 (5)	0.0097 (5)	0.0073 (5)	−0.0021 (4)	0.0006 (4)	0.0000 (4)
Mn2	0.0053 (5)	0.0125 (6)	0.0087 (6)	0.0006 (4)	0.0003 (4)	−0.0009 (4)
As1	0.0043 (4)	0.0072 (4)	0.0043 (4)	−0.0005 (3)	0.0007 (3)	−0.0001 (3)
As2	0.0036 (4)	0.0060 (4)	0.0069 (4)	−0.0002 (3)	0.0007 (3)	0.0007 (3)
O1	0.006 (2)	0.011 (2)	0.010 (2)	0.0021 (17)	0.0015 (17)	0.0034 (19)
O2	0.017 (2)	0.006 (2)	0.012 (3)	0.0009 (18)	0.0022 (19)	0.0023 (19)
O3	0.006 (2)	0.009 (2)	0.016 (3)	0.0021 (17)	0.0009 (18)	0.005 (2)
O4	0.009 (2)	0.009 (2)	0.008 (2)	0.0015 (18)	0.0040 (17)	−0.0008 (18)
O5	0.013 (2)	0.013 (2)	0.006 (2)	−0.0032 (18)	0.0037 (18)	−0.001 (2)
O6	0.017 (3)	0.018 (3)	0.004 (2)	0.002 (2)	−0.0009 (19)	0.004 (2)
O7	0.008 (2)	0.013 (2)	0.016 (3)	−0.0008 (18)	−0.0042 (19)	0.007 (2)
O8	0.006 (2)	0.018 (2)	0.011 (2)	−0.0018 (18)	0.0055 (18)	0.002 (2)

### Geometric parameters (Å, º)

**Table:**

Ba1—O2^i^	2.647 (4)	Mn2—O8^viii^	2.094 (5)
Ba1—O7^ii^	2.682 (5)	Mn2—O4	2.136 (5)
Ba1—O6^iii^	2.687 (5)	Mn2—O5^vii^	2.152 (5)
Ba1—O7	2.741 (5)	Mn2—O3	2.178 (5)
Ba1—O8^iv^	2.853 (5)	Mn2—O7	2.518 (5)
Ba1—O6^v^	2.921 (5)	Mn2—O5^ix^	2.526 (5)
Ba1—O3	3.000 (5)	As1—O6	1.663 (5)
Ba1—O1^v^	3.131 (5)	As1—O1	1.697 (5)
Mn1—O4^vi^	2.052 (5)	As1—O5	1.709 (5)
Mn1—O2^i^	2.108 (5)	As1—O3	1.710 (4)
Mn1—O1^v^	2.179 (4)	As2—O7	1.669 (5)
Mn1—O1^vii^	2.200 (5)	As2—O2	1.677 (5)
Mn1—O3	2.202 (5)	As2—O8	1.677 (4)
Mn1—O5	2.491 (5)	As2—O4	1.698 (5)
Mn1—Mn1^vi^	3.185 (2)		
			
O2^i^—Ba1—O7^ii^	133.63 (15)	O8^viii^—Mn2—O5^ix^	94.05 (18)
O2^i^—Ba1—O6^iii^	74.41 (15)	O4—Mn2—O5^ix^	79.10 (17)
O7^ii^—Ba1—O6^iii^	90.91 (15)	O5^vii^—Mn2—O5^ix^	81.45 (17)
O2^i^—Ba1—O7	127.00 (15)	O3—Mn2—O5^ix^	173.25 (17)
O7^ii^—Ba1—O7	71.27 (16)	O7—Mn2—O5^ix^	94.82 (16)
O6^iii^—Ba1—O7	158.19 (14)	O6—As1—O1	111.1 (2)
O2^i^—Ba1—O8^iv^	144.78 (15)	O6—As1—O5	114.6 (2)
O7^ii^—Ba1—O8^iv^	69.26 (14)	O1—As1—O5	110.1 (2)
O6^iii^—Ba1—O8^iv^	79.72 (15)	O6—As1—O3	109.1 (2)
O7—Ba1—O8^iv^	82.17 (15)	O1—As1—O3	109.0 (2)
O2^i^—Ba1—O6^v^	67.93 (15)	O5—As1—O3	102.5 (2)
O7^ii^—Ba1—O6^v^	152.74 (15)	O7—As2—O2	111.2 (2)
O6^iii^—Ba1—O6^v^	78.74 (16)	O7—As2—O8	114.4 (3)
O7—Ba1—O6^v^	111.37 (14)	O2—As2—O8	109.7 (2)
O8^iv^—Ba1—O6^v^	84.02 (14)	O7—As2—O4	99.8 (2)
O2^i^—Ba1—O3	63.79 (14)	O2—As2—O4	109.1 (2)
O7^ii^—Ba1—O3	94.34 (15)	O8—As2—O4	112.2 (2)
O6^iii^—Ba1—O3	126.26 (14)	As1—O1—Mn1^viii^	121.9 (2)
O7—Ba1—O3	69.23 (14)	As1—O1—Mn1^vii^	125.5 (2)
O8^iv^—Ba1—O3	150.59 (13)	Mn1^viii^—O1—Mn1^vii^	93.34 (18)
O6^v^—Ba1—O3	112.17 (13)	As1—O1—Ba1^viii^	93.03 (18)
O2^i^—Ba1—O1^v^	58.18 (14)	Mn1^viii^—O1—Ba1^viii^	85.45 (14)
O7^ii^—Ba1—O1^v^	146.18 (14)	Mn1^vii^—O1—Ba1^viii^	133.22 (19)
O6^iii^—Ba1—O1^v^	121.73 (13)	As2—O2—Mn1^ix^	117.6 (2)
O7—Ba1—O1^v^	78.33 (13)	As2—O2—Ba1^ix^	141.9 (3)
O8^iv^—Ba1—O1^v^	121.31 (13)	Mn1^ix^—O2—Ba1^ix^	100.46 (18)
O6^v^—Ba1—O1^v^	54.34 (13)	As1—O3—Mn2	127.5 (2)
O3—Ba1—O1^v^	60.47 (12)	As1—O3—Mn1	98.5 (2)
O4^vi^—Mn1—O2^i^	102.96 (19)	Mn2—O3—Mn1	127.0 (2)
O4^vi^—Mn1—O1^v^	99.75 (17)	As1—O3—Ba1	104.3 (2)
O2^i^—Mn1—O1^v^	82.98 (19)	Mn2—O3—Ba1	101.98 (16)
O4^vi^—Mn1—O1^vii^	84.93 (19)	Mn1—O3—Ba1	88.38 (16)
O2^i^—Mn1—O1^vii^	167.89 (17)	As2—O4—Mn1^vi^	127.8 (3)
O1^v^—Mn1—O1^vii^	86.66 (18)	As2—O4—Mn2	99.8 (2)
O4^vi^—Mn1—O3	166.2 (2)	Mn1^vi^—O4—Mn2	121.3 (2)
O2^i^—Mn1—O3	88.15 (19)	As1—O5—Mn2^vii^	122.2 (3)
O1^v^—Mn1—O3	89.68 (17)	As1—O5—Mn1	88.39 (19)
O1^vii^—Mn1—O3	85.54 (18)	Mn2^vii^—O5—Mn1	97.19 (19)
O4^vi^—Mn1—O5	104.50 (16)	As1—O5—Mn2^i^	129.8 (3)
O2^i^—Mn1—O5	79.89 (17)	Mn2^vii^—O5—Mn2^i^	98.55 (17)
O1^v^—Mn1—O5	152.85 (16)	Mn1—O5—Mn2^i^	116.29 (18)
O1^vii^—Mn1—O5	107.30 (17)	As1—O6—Ba1^iii^	137.3 (3)
O3—Mn1—O5	68.96 (16)	As1—O6—Ba1^viii^	101.6 (2)
O8^viii^—Mn2—O4	150.46 (18)	Ba1^iii^—O6—Ba1^viii^	101.26 (16)
O8^viii^—Mn2—O5^vii^	116.23 (18)	As2—O7—Mn2	87.0 (2)
O4—Mn2—O5^vii^	91.36 (18)	As2—O7—Ba1^ii^	132.7 (2)
O8^viii^—Mn2—O3	92.31 (19)	Mn2—O7—Ba1^ii^	96.77 (16)
O4—Mn2—O3	96.40 (18)	As2—O7—Ba1	116.9 (2)
O5^vii^—Mn2—O3	93.72 (19)	Mn2—O7—Ba1	100.85 (16)
O8^viii^—Mn2—O7	85.61 (17)	Ba1^ii^—O7—Ba1	108.73 (16)
O4—Mn2—O7	66.64 (17)	As2—O8—Mn2^v^	127.9 (3)
O5^vii^—Mn2—O7	157.97 (16)	As2—O8—Ba1^iv^	116.1 (2)
O3—Mn2—O7	87.90 (17)	Mn2^v^—O8—Ba1^iv^	102.60 (17)

Symmetry codes: (i) *x*, *y*+1, *z*; (ii) −*x*, −*y*+1, −*z*; (iii) −*x*, −*y*+2, −*z*; (iv) −*x*+1, −*y*+1, −*z*; (v) *x*+1, *y*, *z*; (vi) −*x*+1, −*y*+2, −*z*+1; (vii) −*x*, −*y*+2, −*z*+1; (viii) *x*−1, *y*, *z*; (ix) *x*, *y*−1, *z*.
