# Supplementary material for: Crystal structure of BaMn2(AsO4)2 containing discrete [Mn4O18]28− units
Source: Acta Crystallogr E Crystallogr Commun. 2017 Nov 14;73(Pt 12):1855–60. doi: 10.1107/S2056989017016152 (PMC5730239; doi:10.1107/S2056989017016152)
Supplement: Supplementary file 3 [file e-73-01855-sup3.pdf]

**Supplementary Table 1.**

Overview of isotypic $AM_2(XO_4)_2$ compounds with $A$ = Ba, Sr; $M$ = Mn, Co, Ni, and $X$ = As, P.

| Compound | Space group | a (Å)<br>α (°) | b (Å)<br>β (°) | c (Å)<br>γ (°) | $d_{M\text{---}M}$ (Å)<br>∠$M$1-O-$M$1 (°) | Configuration of $M$1 and $M$2 polyhedra | |
|---|---|---|---|---|---|---|---|
| $BaMn_2(AsO_4)_2$ | $P\bar{1}$ | 5.7981(12)<br>109.75(3) | 7.0938(14)<br>100.42(3) | 9.817(2)<br>98.40(3) | 3.185(2)<br>93.34(18) | $M1O_{(5+1)}$ | $M2O_{(4+2)}$ |
| $\beta$-$SrCo_2(PO_4)_2$ | $P\bar{1}$ | 5.503(3)<br>110.235(16) | 6.703(3)<br>101.15(3) | 9.238(5)<br>98.29(2) | 3.049(2)<br>91.64(13) | $M1O_{(5+1)}$ | $M2O_{(4+2)}$ |
| $SrCo_2(AsO_4)_2$ | $P\bar{1}$ | 5.713(7)<br>110.48(9) | 6.901(7)<br>101.43(6) | 9.417(11)<br>99.13(5) | 3.043<br>92.13 | $M1O_{(5+1)}$ | $M2O_{(4+2)}$ |
| $SrNi_2(PO_4)_2$ | $P\bar{1}$ | 5.468(1)<br>110.58(1) | 6.667(1)<br>100.87(1) | 9.156(1)<br>98.01(1) | 2.993(1)<br>92.06 | $M1O_6$ | $M2O_{(5+1)}$ |
